# Characterization of anti-drug antibody responses to the T-cell engaging bispecific antibody cibisatamab to understand the impact on exposure

**DOI:** 10.3389/fimmu.2024.1406353

**Published:** 2024-05-31

**Authors:** Gregor P. Lotz, Achim Lutz, Meret Martin-Facklam, Andre Hansbauer, Eginhard Schick, Ekkehard Moessner, Michael Antony, Thomas Stuchly, Maria Viert, Ralf J. Hosse, Anne Freimoser-Grundschober, Christian Klein, Martin Schäfer, Mirko Ritter, Kay-Gunnar Stubenrauch

**Affiliations:** ^1^ Roche Pharma Research and Early Development, Roche Innovation Center Munich, Penzberg, Germany; ^2^ Roche Pharma Research and Early Development, Roche Innovation Center Basel, Basel, Switzerland; ^3^ Roche Pharma Research and Early Development, Roche Innovation Center Zurich, Schlieren, Switzerland; ^4^ Roche Diagnostics GmbH, Antibody Development Technologies, Penzberg, Germany

**Keywords:** PK, exposure, immunogenicity, ADA, T cell engager

## Abstract

An appropriately designed pharmacokinetic (PK) assay that is sensitive for anti-drug antibody (ADA) impact on relevant exposure is an alternative strategy to understand the neutralizing potential of ADAs. However, guidance on how to develop such PK assays and how to confirm the functional ADA impact on exposure is missing. Here, the PK assay of a T-cell-engaging bispecific antibody, cibisatamab, was developed based on its mechanism of action (MoA). Using critical monoclonal anti-idiotypic (anti-ID) antibody positive controls as ADA surrogates, the impact on exposure was evaluated pre-clinically. In a phase I clinical trial (NCT02324257), initial data suggest that the combination of ADA and PK assays for correlation of the ADA response with cibisatamab exposure. To understand the neutralizing potential of patient-derived ADAs on drug activity, advanced ADA characterization has been performed. Structural binding analysis of ADAs to antibody domains of the drug and its impact on targeting were assessed. For this purpose, relevant patient ADA binding features were identified and compared with the specific monoclonal anti-ID antibody-positive controls. Comparable results of target binding inhibition and similar impacts on exposure suggest that the observed reduction of Cmax and Ctrough levels in patients is caused by the neutralizing potential of ADAs and allows a correlation between ADA response and loss of exposure. Therefore, the described study provides important functional aspects for the development of an appropriately designed PK assay for bispecific antibodies as an alternative option towards understanding the neutralizing ADA impact on exposure.

## Introduction

It is critical during the clinical development of biotherapeutics to generate precise pharmacokinetic (PK) data to understand the relationship between exposure and pharmacodynamic (PD) response, safety, and efficacy as a prerequisite for dose and schedule selection (1). The analysis of relevant drug exposure becomes particularly important when patients demonstrate an anti-drug antibody (ADA) response during treatment. In such cases, it is necessary to understand the impact of ADAs on drug exposure, which is ideally correlated with the corresponding PD effect.

A key aspect of assessing ADA impact on exposure is the combination of ADA analysis with PK analysis using a PK assay that is sensitive for ADA impact on the pharmacologically relevant drug exposure. The combination of both assay data allows the correlation of drug exposure with the ADA response for a given patient. A prerequisite for the development of a PK assay analyzing relevant drug exposure is the generation of specific reagent assay tools such as the target antigen or, alternatively, anti-idiotypic (anti-ID) antibodies directed to epitopes of the binding sites that are involved in target binding (2). Using antigen-reagents or appropriate anti-ID antibodies as reagents ensures coverage of the united functionalities of the drug and allows the development of a PK assay based on its mechanism of action (MoA).

Another important step during the development of the PK assay is an adequate evaluation of the impact of ADAs on exposure. However, it is difficult to address the ADA impact on drug exposure pre-clinically due to a lack of patient-derived ADA characteristics. At present, the widely accepted standard is the use of ADA surrogate tools often generated in animal models as positive controls for the development of ADA assays and the pre-clinical evaluation of ADA impact on clinical PK assays (3). What is often missing is the retrospective validation of the ADA positive controls to confirm that they are comparable to patient-derived ADAs, thus evaluating and confirming that the PK assay is indeed sensitive to the impact of ADAs on exposure.

In this study, the development of a PK assay for the bispecific T-cell engager cibisatamab based on its MoAs is described, and its performance in clinical trials is shown. The ADA impact on exposure was evaluated using ADA-positive controls and compared with functional patient-derived ADA binding to the drug, and its interference with target antigen binding was demonstrated. Based on the patient’s ADA binding features, appropriate anti-ID ADA-positive controls with similar binding features were identified and selected to validate the PK assay. Comparable results of impact on exposure by patient-derived ADAs and selected ADA positive controls suggest that the observed reduction of Cmax and Ctrough level in patients is caused by neutralizing ADAs and allows a correlation between ADA response and loss of exposure. Taken together, the results of this study may provide criteria to be considered for the development of appropriate PK assays to understand the impact of potential neutralizing ADAs.

## Results

### Development of a mechanism of action-based PK assay that is sensitive for ADA impact to analyze relevant exposure

**Figure 1:**
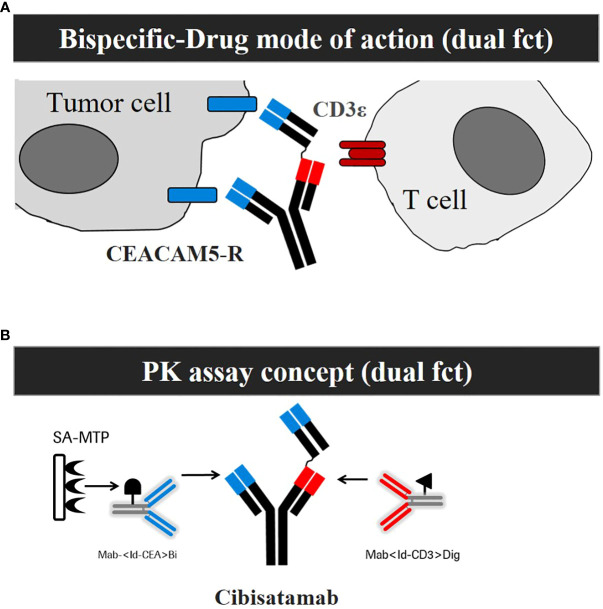
Development of a mechanism of action (MoA) based Pharmacokinetic (PK) assay that is sensitive for ADA impact on exposure. **(A)** Schemata of the MoA of cibisatamab with its dual functionality (dual fct). **(B)** Schemata of the PK Assay concept with its dual functionality (dual fct).

The design of the assay format to develop an appropriate PK assay is a key prerequisite to enable the detection of relevant drug exposure (4–6). The first important aspect to consider is the MoA of the drug. The MoA of cibisatamab is based on the simultaneous binding of cibisatamab to CEA/CEACAM5 on tumor cells and to the CD3ε chain on T cells, which results in T-cell activation and subsequent tumor cell killing (7, 8). This dual binding functionality was implemented for the development of a MoA-based PK assay (**Figures 1A, B**). A second important aspect of assay development is the production and characterization of high-quality reagents (2). To mimic the MoA, monoclonal anti-ID antibodies were generated, purified, and characterized for each specific functional binding site of cibisatamab (**Figure 1B**). These anti-ID antibody reagents directed to the target-binding relevant complementarity-determining regions (CDRs) of the anti-CEA and to the anti-CD3 domains of cibisatamab allow the quantification of drug concentration, exposing its free binding moieties to both targets along with an ultrasensitive detection of <1 ng/ml. In **Table 1**, inter-assay statistics of standards and quality control drug concentrations result in accuracy and precision assay performance within the acceptance criteria during validation and the clinical phase of study I.

**Table 1:** Determination of high accuracy and precision of a MoA-based PK assay.

Cibisatamab PK assay validation					
**Dynamic range**	**Sensitivity**	**Inter-assay statistics (Cals; n=20)**		**Inter-assay statistics (QC samples; n=40)**	
		**Accuracy**	**Precision**	**Accuracy**	**Precision**
0.925–120 ng/mL	0.925 ng/mL	98.1%–102.9%	≤ 4.4% CV	94.8%–100.0%	≤ 12.3% CV
Cibisatamab sample analysis (FiH Study)					
**Inter-assay statistics (Cals; n=256)**		**Inter-assay statistics (QC samples; n=512)**			
**Accuracy**	**Precision**	**Accuracy**		**Precision**
99.6%–101.0%	≤ 3.4% CV	94.4%–100.0%		≤ 12.0% CV

Assay parameters of the MoA-based PK assay demonstrate accurate and precise performance of calibration (Cals) and quality control (QC) samples of recovery and CV values within acceptance criteria during the validation and FiH study.

To evaluate the ADA impact on target-binding competent drug exposure, the recovery of a constant cibisatamab serum concentration (120 ng/ml) was analyzed in the absence (control) and presence of different ADA positive control concentrations (10, 100, and 1000 ng/ml) containing an equimolar mix of two monoclonal anti-ID antibodies, one directed to the anti-CEA and one directed to the anti-CD3 domain (**Supplementary Figure 1A, C**). Each monoclonal anti-ID antibody had comparable binding parameters. Fast association and slow dissociation kinetics with comparable affinities (KD 1 nM to anti-CD3 and 0.64 nM to anti-CEA), respectively, were measured using surface plasmon resonance (not shown) and biolayer interferometry analysis (**Supplementary Figure 1B**/sensogram).

In **Supplementary Figure 1C**, keeping the cibisatamab serum concentration constant at 120 ng/ml, the anti-ID ADA control mix at a concentration of 10 ng/ml reduced the recovery of the drug slightly by ~20%. At a concentration of 100 ng/ml anti-ID ADA PCs, a strong reduction in recovery >90% was observed, suggesting that comparable molar concentrations of anti-ID ADA positive controls and cibisatamab led to a significant decrease in the target-binding-competent drug concentration. Consequently, an anti-ID ADA positive control concentration that is ~10-fold higher results in a complete loss of target-binding-competent drug detection (**Supplementary Figure 1C**). These data suggest that the MoA-based PK assay detects critical target-binding-competent drug exposure and is sensitive to interference by binding of anti-ID ADA positive controls.

**Figure 2:**
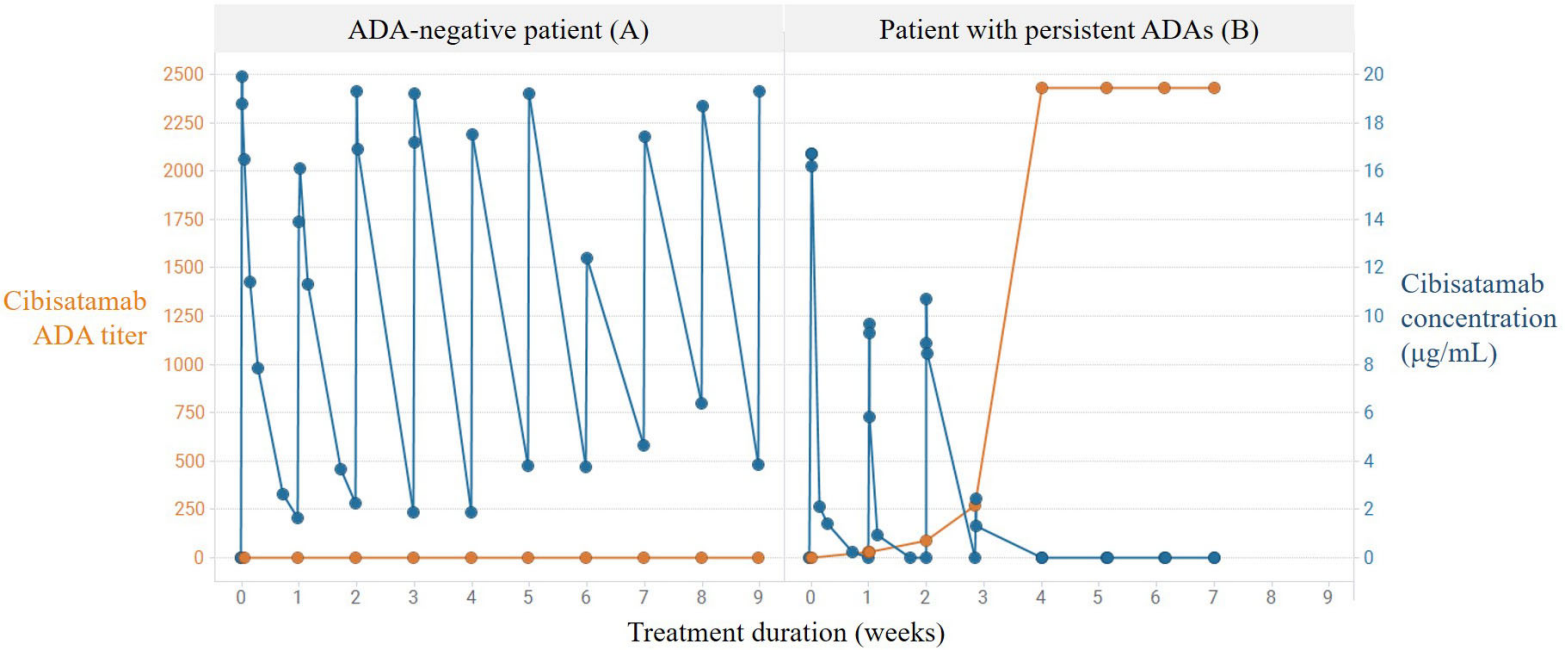
Correlation of persistent ADA response and loss of exposure using MoA-based PK assay. Increase in cibisatamab ADA titer and associated decrease in active cibisatamab exposure (patient B) in contrast to ADA negative patient with maintained active exposure (patient A).

To prove the concept of the PK assay, the impact of patient-related ADA responses to cibisatamab exposure was evaluated. In **Figure 2**, the ADA titer over time was compared with the drug exposure data in one ADA-negative patient (patient A) and in one patient (patient B) with a persistent ADA response. In patient A, who did not develop ADAs, the detectable drug concentrations of cibisatamab were maintained over time, whereas in patient B, a significant reduction of the detectable drug concentration was already observed at the onset of an ADA response after week 1, resulting in a complete loss of exposure from week 4 onwards and a persistently high ADA titer. These data indicate that an increase in cibisatamab ADA titer is associated with a decrease in target-binding competent cibisatamab exposure (patient B), in contrast to an ADA-negative patient where drug exposure is maintained over time (patient A). These data confirm that the use of a MoA-based PK assay allows the correlation of patient-derived ADA responses to exposure.

### Persistent patient-derived ADA response dominantly consists of IgG isotypes and is directed to the anti-CD3 domain, leading to relevant loss of exposure

To better understand the neutralizing potential of the patient-derived ADAs for functional binding and their impact on exposure, an advanced ADA response characterization was performed. Samples of 10 patients were further analyzed to determine (a) the specific ADA-reactive drug domain using an ADA domain detection assay (9, 10) (**Supplementary Figure 2**) and (b) the isotype of the ADA response using ADA immune-complex assays for IgM and IgG detection (11–13) (**Supplementary Figure 3**). Patient serum samples taken 4 weeks after ADA onset were selected and analyzed with domain detection ELISAs specific for ADA detection in the anti-CEA domain or anti-CD3 domain (**Table 2**). In all 10 samples, a strong signal towards the anti-CD3 was detected, whereas only in 3/10 samples, a weak signal towards the anti-CEA domain slightly above the cut point (CP: 0.04 OD) was detected. In **Supplementary Figure 3**, a characteristic ADA response detected in one patient with cibisatamab treatment is shown. At ADA onset, IgM triggered the initial response with a lower titer, whereas at a later time point, the response was mainly determined by the ADA-IgG response with a high titer alongside loss of drug exposure. At 4 weeks after ADA onset (**Supplementary Figure 3**, black arrow), the IgG detection was saturated, the IgM detection was below the cut point, and exposure was lost. Similarly, in all ten selected patient samples collected 4 weeks after ADA onset, the ADA immune-complex analysis using a FcyR1-based detection reagent indicated a strong IgG-related ADA response but hardly any IgM response (**Table 3**). There is no drug that could be detected using the MoA-based PK assay at that time point in all 10 patient samples (**Table 3**). These data suggest that the ADA response in these patients leading to significant loss of exposure was caused by ADA-IgG and mainly directed to the anti-CD3 domain at 4 weeks after ADA onset.

**Table 2:** Patient-derived ADAs are mainly directed to the anti-CD3 domain at 4 weeks after ADA onset.

Patient (Samples taken 4 weeks after ADA onset)	Total ADA assay (Screening)	ADA CD3-Domain assay	ADA CEA-domain assay
1	Positive	+++	Negative
2	Positive	+++	Negative
3	Positive	++	Negative
4	Positive	+++	Negative
5	Positive	+++	+
6	Positive	+++	Negative
7	Positive	++	Negative
8	Positive	+++	+
9	Positive	+++	Negative
10	Positive	+++	+
Strong: +++ (> OD 1.0) Medium: ++ (> OD 0.3) Low: + (> CP) Negative: (< CP)	Cut Point (OD): 0.04; baseline control mean: 0.035		

Ten patient serum samples collected 4 weeks after ADA onset were analyzed with anti-CD3 and anti-CEA domain binding assays.

**Table 3:** The ADA response leading to loss of exposure at the end of infusion to the signal below LoQ is mainly caused by ADA IgG isotypes at 4 weeks after ADA onset.

Patient (Samples taken 4 weeks after ADA onset)	Total ADA assay (Screening)	ADA IgG assay	ADA IgM assay	Loss of exposure (PK assay)
1	Positive	+++	Negative	Yes
2	Positive	+++	Negative	Yes
3	Positive	+++	Negative	Yes
4	Positive	+++	Negative	Yes
5	Positive	+++	Negative	Yes
6	Positive	+++	Negative	Yes
7	Positive	+++	Negative	Yes
8	Positive	+++	Negative	Yes
9	Positive	+++	+	Yes
10	Positive	+++	Negative	Yes
Strong: +++ (> OD 1.5) Medium: ++ (> OD 0.75) Low: + (>CP) Negative: (<CP)	Individual patient-specific Cut Point (OD) IgG and IgM assay: double pre-dose patient sample signal (blank)			Signal below the LoQ

### Advanced characterization of anti-ID antibodies as ADA-positive controls

The deeper characterization of patient-derived ADAs 4 weeks after onset determined IgG as the major isotype and anti-CD3 domain binding specificity to cibisatamab. However, whether ADAs were anti-ID with neutralizing potential was not yet clear. To explore these questions, first, anti-ID antibodies were further evaluated to establish adequate ADA-positive controls for advanced binding and neutralizing studies. Four purified, monoclonal anti-IDs were selected to analyze their specific binding to the anti-CD3 domain of cibisatamab individually (**Supplementary Figure 4**). All four anti-IDs showed similar binding features to the anti-CD3 domain of cibisatamab, including a strong signal using an ELISA assay (**Supplementary Figure 4A**) and fast association and slow dissociation kinetics using biolayer interferometry analysis (**Supplementary Figure 4B**).

**Figure 3:**
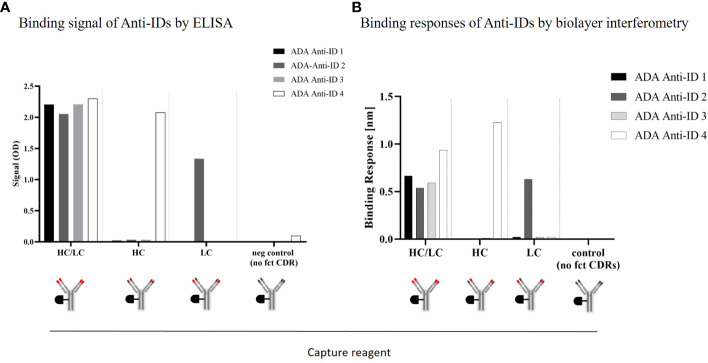
ADA anti-CD3/CDR domain specificity characterization: Binding characteristics of four different monoclonal anti-idiotypic antibodies to modified anti-CD3 domains analyzed by ELISA **(A)** and biolayer interferometry **(B)**. HC, heavy chain; LC, light chain; CDR, complementarity-determining region.

Based on structural data, the heavy chain (HC) plays an important role in recognizing the CD3ϵ receptor (data not shown). To understand if the anti-IDs were directed to the CDRs of the HC or to the CDRs of the light-chain (LC) of the anti-CD3 domain, specific anti-CD3 domain constructs were engineered, purified, and used as capture reagents for CDR-specific domain detection assays (**Supplementary Figure 5**). The specific anti-CD3 domain constructs had either functional CDRs in the V-domain of the heavy chain with germline CDRs in the V-domains of the light chain or vice versa. Corresponding controls, such as a construct with both functional CDRs in the antibody paratope, were used as a positive control, or germline CDR sequences in the antibody paratope were used as a negative control. In **Figure 3A**, each anti-ID antibody was able to bind to the HC/LC positive control but did not bind to the negative control construct (germline in HC/LC) using an ELISA assay. Interestingly, only one anti-ID (anti-ID 4) bound to the anti-CD3 domain construct with functional CDRs in the HC, whereas the other three anti-ID antibodies did not. Similarly, using the anti-CD3 domain constructed with the functional CDRs of the LC, another anti-ID antibody (ADA anti-ID 2) was able to bind, and the other three were not. These results were additionally confirmed with an orthogonal binding readout using biolayer interferometry (**Figure 3B**). In summary, all four monoclonal anti-ID antibodies with similar binding affinities showed strong binding to fully functional CDRs. The anti-ID antibodies were directed to distinct CDR epitopes in the anti-CD3 variable domains and, consequently, might have different neutralizing interference potentials from cibisatamab binding to its CD3ϵ antigen receptor target.

### Anti-ID antibodies inhibit receptor antigen-target engagement of an anti-CD3 IgG drug in a CD3-receptor reporter cell assay

**Figure 4:**
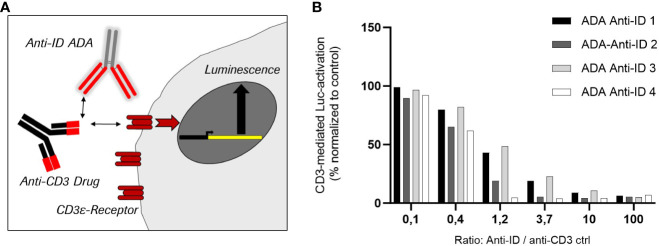
Inhibition of cell-based target CD3 receptor mediated luciferase activity by four different monoclonal anti-idiotypic antibodies as potential ADA positive controls. **(A)** Schemata of CD3ε-receptor mediated reporter cell line using anti-CD3 antibodies as stimulating control. **(B)** Effect of the four anti-idiotypic antibodies in CD3-mediated luciferase activation.

To analyze the neutralizing potential of the four selected anti-ID antibodies as relevant ADA positive controls, a CD3ϵ antigen receptor-specific reporter cell line was used. Stimulation of the highly expressed CD3ϵ antigen receptors on the cell surface activates a reporter gene expressing luciferase. The corresponding luminescence signal correlates with the CD3ε-mediated luciferase activation (**Figure 4**). To enable the best possible read-out, an anti-CD3 IgG antibody was used as a stimulating control for maximal signal response. This anti-CD3 control contains the same binding sequence in the CDR as in the cibisatamab anti-CD3 domain. The interference of the four anti-IDs with CD3ε-mediated activation was tested by pre-incubation in different ratios with an anti-CD3 IgG control. All four monoclonal anti-ID antibodies abolished CD3ε-mediated cell activation completely when the anti-ID concentration was in at least 10-fold excess over the anti-CD3 IgG control concentration (anti-ID-control ratio 10 or 100) (**Figure 4**). At a ratio of 1.2, two anti-ID antibodies (Anti-ID 2; Anti-ID 4) still abolished the signal significantly, from 80% to 100%, and two anti-ID antibodies (Anti-ID 1; Anti-ID 3) showed a reduction of 50% of the signal. Although not all anti-ID antibodies at equimolar concentrations to the anti-CD3 IgG control suppressed CD3ϵ-mediated activation equally, each selected anti-ID that bound to functional binding sites of the anti-CD3 drug domain strongly interfered with CD3ε receptor antigen binding. These data demonstrate that all anti-ID antibodies are valid ADA-positive controls with a neutralizing effect on drug-antigen target binding and on CD3ε-mediated signaling.

### Patient-derived ADAs are anti-ID antibodies and predominantly directed at the heavy-chain CDRs of the anti-CD3 domain

The ADA positive controls used for the evaluation of the PK assay to measure if the assay is sensitive for ADA impact on free-drug exposure were anti-IDs and able to interfere with antigen-target binding. To understand if patient-derived ADAs are also anti-ID antibodies and directed to functional CDRs, the same-engineered anti-CD3 domain constructs with modified functional CDR (HC, LC, or both) were used to analyze the same 10 patient samples, taken 4 weeks after ADA onset.

In **Table 4**, all 10 patient samples were shown to be ADA positive using an ADA screening bridging ELISA (total ADA/column 2). In addition, all samples were detected ADA-positive using either the anti-CD3 domain capture (**Table 4**/Column 3) or the construct with full functional CDRs (**Table 4**/Column 4), suggesting that the patient-derived ADAs were anti-ID and directed to the anti-CD3 domain of cibisatamab. Baseline pre-dose samples (**Table 4**/Column 1) were instead negative in all 10 samples. A second control using constructs with unrelated CDRs (**Table 4**/Column 7) showed 4/10 positive, but all signals were borderline and only slightly above the very low cut point of 0.032 OD, indicating that these signals were rather falsely positive than real positive signals. Interestingly, 9/10 patient samples showed reactivity to the anti-CD3 construct with functional CDRs of the HC (**Table 4**/column 5), whereas only 5/10 have shown a binding signal towards the anti-CD3 construct with functional LC CDRs (**Table 4**/column 6). The signal strength of the HC anti-CD3 construct was high (> 1.0 OD), whereas the five positive samples in the LC anti-CD3 construct were borderline signals slightly above the cut point. These results indicate that patient-derived ADAs were anti-ID antibodies and mainly directed to the HC of the cibisatamab anti-CD3 domain rather than to the LC, suggesting a specific, immune-dominant epitope on the HC.

**Table 4:** ADAs to the anti-CD3 domain are anti-idiotypic and directed dominantly to the CDRs of the heavy chain.

	Column 1	Column 2	Column 3	Column 4	Column 5	Column 6	Column 7
Patient (Samples 4 weeks after ADA onset)	Pre-dose baseline sample (Screening)	Total ADA assay ADA screen	Anti-CD3 domain	Anti-CD3 full CDR	Anti-CD3 HC-CDR	Anti-CD3 LC-CDR	Unrelated CDR germline control
ADA +/sample	0/10	10/10	10/10	10/10	9/10	5/10	4/10
1	Negative	+++	+++	+++	++	+ (0.078)	+ (0.077)
2	Negative	+++	+++	+++	++	+ (0.034)	Negative
3	Negative	+	+	+	Negative	Negative	Negative
4	Negative	+++	+++	+++	+	Negative	Negative
5	Negative	++	++	+++	+	Negative	Negative
6	Negative	++	++	+++	+	Negative	Negative
7	Negative	++	++	+++	++	+ (0.039)	+ (0.042)
8	Negative	+++	+++	++	+	Negative	Negative
9	Negative	+++	+++	+++	+++	+ (0.042)	+ (0.04)
10	Negative	+++	+++	+++	+++	+ (0.064)	+ (0.062)
**Cut point (CP)**	CP: 0.04	CP: 0.04	CP: 0.04	CP: 0.04	CP: 0.034	CP: 0.029	CP: 0.032

Strong: +++ (> OD 1.0); Medium: ++ (> OD 0.3); Low: + (>CP); Negative: (<CP).

### Patient-derived ADAs strongly inhibit receptor antigen-target engagement of an anti-CD3 IgG drug in a CD3-receptor cell assay

**Figure 5:**
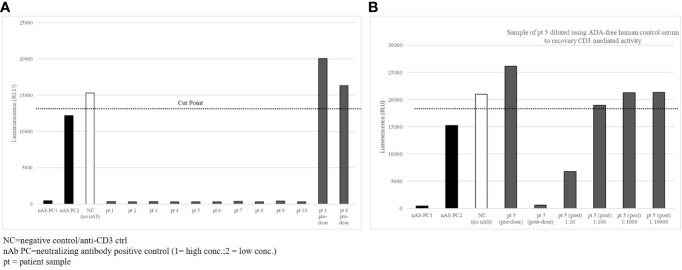
Patient-derived ADAs inhibit CD3ε-receptor mediated activation. **(A)** 10 selected patient samples with high titer ADA detection were tested in the CD3ε receptor mediated reporter assay with a complete loss of signal **(B)** Sample of patient 5 diluted in ADA-free human control serum to recovery CD3ε mediated activity.

To demonstrate the neutralizing interference of the CD3-specific ADA response in patients, the CD3ϵ-specific reporter cell line was used with the same ADA-positive samples taken 4 weeks after the onset of ADA in the selected ten patients. As expected, the anti-CD3 IgG antibody control mediated strong luminescence signals in pre-dose samples (**Figure 5**, pt1 and pt8 predose) and control samples with a human serum pool (**Figure 5**, NC). Two concentrations of the anti-ID antibody mix were used as neutralizing positive controls (nAb PC1, nAb PC2). All treatment-induced, patient-derived ADAs inhibited the signal completely (**Figure 5A**, pt1–10). These data indicate that patient-derived ADAs interfered with drug binding to the CD3ϵ receptor and elicited the neutralizing potential of ADAs similar to the tested monoclonal anti-ID antibodies.

ADA inhibition of drug-target binding is dependent on the molar ADA-drug ratio. To demonstrate this in patient samples of the cibisatamab study, samples of patient 5 were diluted with ADA-free serum by maintaining a constant stimulating anti-CD3 IgG control concentration to evaluate if full recovery of the signal could be reached. Before diluting the samples, the anti-CD3 IgG control activated the Jurkat T cells in the reporter assay in the pre-dose sample, whereas the post-dose sample showed complete inhibition (**Figure 5B**, pre- and post-dose). ADA dilutions of 1:10 and 1:100 in ADA-free serum allowed increased recovery of the signal, and dilutions of 1:1000 and 1:10000 in ADA-free serum brought the luciferase signal back to full recovery. In total, the data suggest that ADAs collected 4 weeks after the onset of an ADA response were directed to the functional target binding sites of the CD3 domain of cibisatamab and neutralized drug target binding to CD3ε receptors.

### Exposure to cibisatamab is impacted by ADAs in the phase I study using the MoA-based PK assay

**Figure 6:**
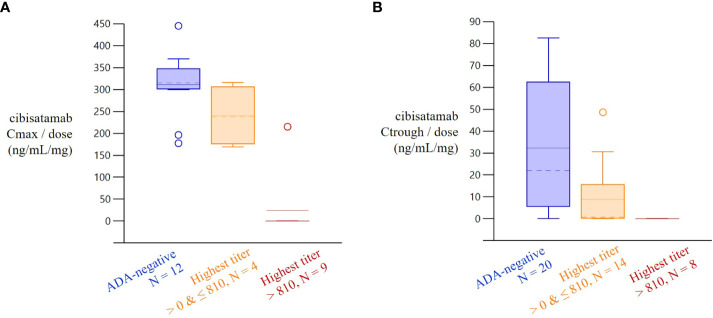
Increase in Cibisatamab ADA titer and associated decrease in cibisatamab exposure. Dose-normalized cibisatamab Cmax and Ctrough by highest ADA-titer 4 weeks after treatment start. In patients with high ADA-titers (>810), both Cmax and Ctrough/min were relevantly decreased. Patients with low ADA titers (<810) tended to have Cmax values that were not relevantly smaller than in ADA-negative patients, although decrease in Ctrough/min may be more pronounced.

The MoA-based PK assay used to detect the cibisatamab concentration has been demonstrated to be sensitive for potential neutralizing anti-ID ADA responses. To investigate the correlation in the clinic, PK and ADA data were collected in a phase I dose-escalation and expansion study of cibisatamab monotherapy (NCT02324257) in patients with advanced CEA-positive solid tumors. An increase in cibisatamab ADA titers was associated with a decrease in target-binding competent cibisatamab exposure (**Figure 6**). Usually, the concentration prior to the next infusion (Ctrough) decreases first, followed by a decrease in the maximum concentration after the infusion (Cmax). Patients with low ADA titers (<810) tended to have Cmax values that were not significantly lower than in ADA-negative patients, although a decrease in Ctrough may have been more pronounced. Strikingly, in patients with high ADA-titers (>810), both Cmax and Ctrough were significantly decreased, even to a complete loss of exposure, i.e., no target-binding-competent cibisatamab is detectable in serum directly, even shortly after dosing, when cmax is expected.

## Discussion

To inform decision-making during clinical development, it is critical to rely on exposure data that can be correlated with efficacy and safety data. Therefore, it is important to understand which drug species (e.g., total, target binding competent, complexed) are being measured (4–6, 14). This process is even more important when multiple functionalities and binding sites are combined in one drug (15). It is thus essential to consider and involve all functionalities of the drug in the PK assay(s) to be used for sample analysis.

This study describes the development of a MoA-based PK assay of the T-cell engaging bispecific antibody cibisatamab using anti-ID antibodies to allow analysis of functionally relevant target-binding-competent drug exposure. To mimic the MoA appropriately, it is fundamental to select high-quality reagents to enable such a development and to transfer the dual intra-dependent functionality of the drug molecule into the assay format design. Here, it is necessary to prove the capability of the anti-ID reagent to bind to relevant drug binding sites involved in target interaction (2). Another important aspect of a MoA-based assay is to capture information about the molecular integrity of the drug during analysis and to exclude the detection of potential structurally misfolded, dysfunctional drugs. Long-term supply of these reagents with high quality, low batch-to-batch variability, and high production yield are further advantages of using anti-ID reagent tools (2).

Immediately after the MoA-based PK assay is developed, it is critical to evaluate the sensitivity of the PK assay to potential biological interferences on drug detection, e.g., due to bound ADAs (6). In this study, multiple ADA-positive controls were characterized in depth to prove their validity to interfere with the drug’s functionality and its drug-target binding. It was demonstrated that all ADA-positive controls inhibit drug-CD3ϵ target-mediated activation and, therefore, indicate a loss of drug function. This deeper pre-clinical evaluation of appropriate ADA positive controls on PK assay performance allowed retrospective translation of clinical exposure data along with ADA response data. Indeed, a broader evaluation of the clinical data in the phase I study suggests a strong correlation between high-titer ADAs and a significant loss of cibisatamab exposure and proves the concept of a MoA-based PK assay performance to measure target binding competent drug exposure.

The deeper characterization of ADA-positive controls was an advantage in identifying relevant patient ADA-binding features. Specific CDRs of cibisatamab were identified as being involved in the binding of ADA-positive controls and also in the binding of patient ADAs. CDRs of the heavy chain seem to play an essential role in terms of ADA binding. Interestingly, CDRs of the heavy chain were critical for CD3ϵ receptor antigen binding (data not shown), suggesting that ADAs (controls and patient ADAs) interfere with functional antigen binding. Indeed, the one anti-ID ADA PC (anti-ID 4) that specifically reacted with the CDRs of the HC was the most efficient inhibitor of CD3ε receptor-mediated activation.

Domain characterization data shows that patient-derived ADAs were anti-ID antibodies and dominantly directed to the CDRs of the anti-CD3 domain when collected 4 weeks after ADA onset. An anti-ID ADA response to drug-specific CDRs interferes with drug target binding and results in a neutralizing impact on drug function (16). ADA isotype characterization further demonstrated that the evaluation of the ADA responses revealed a class switch from an initial IgM response to a stronger (high titer) IgG response as described for classical immune responses (17). Maturation to the ADA IgG isotype response in these patients might be an indication of a more drug-specific epitope along with an increased ADA affinity for the drug. The characterized ADA positive controls used in this study seem to be directed to such an epitope and serve not only as appropriate and important tools to understand the neutralizing impact on PK exposure analyses but can also be used for further epitope characterization and/or complex analyses. The identification of specific B-cell epitopes via ADA binding evaluation combined with screening of potential drug-sequence-related T-cell epitopes might be an interesting evaluation to better understand the T-B-cell interaction of the ADA immune response in cibisatamab, similar to what was demonstrated in natalizumab (16).

Another advantage of using a MoA-based PK assay and understanding the neutralizing impact of ADAs is the quantitative free-drug concentration analysis over time. These quantitative PK analyses are more informative to support decision-making during clinical development than neutralizing ADA assays (nAb assays), which usually only have qualitative read-outs and no information about whether the remaining functional target-binding competent drug is still in circulation (18, 19).

In summary, the development of an appropriate MoA-based PK assay called for deeper exploration of appropriate assay reagent tools. The retrospective control analysis with clinical data and an advanced patient-derived ADA characterization for pre-clinical assay evaluation supplemented the essential proof of assay performance. The availability of correct target-binding, competent exposure data sensitive to ADAs is fundamental for adequate correlation with safety and efficacy to support informed decision-making during drug development. Target-binding PK assays might be used in lieu of qualitative nAb assays.

## Materials and methods

### Antibodies and reagents

The test compound cibisatamab is a bispecific therapeutic antibody in a head-to-tail 2:1 format and is described in previous publications (7, 8).

Murine monoclonal anti-ID antibodies against CDRs of cibisatamab mAb<Id-mAb<H-CEA>>IgG-Bi as a biotin-labeled capture reagent and mAb<Id-mAb<H-CD3>>IgG-Dig as a digoxigenin-labeled detection reagent was produced by Roche Diagnostics GmbH, Penzberg, Germany, for the development of the PK assay.

Cibisatamab was labeled with biotin or digoxigenin by Roche Diagnostics GmbH for the development of the ADA-bridging ELISA assay.

Murine monoclonal anti-ID antibodies 1–4 as ADA positive controls, specifically generated against CDRs of the anti-CD3 domain of cibisatamab, were produced and labeled by Roche Diagnostics GmbH, Germany.

Specific domains of cibisatamab and anti-CD3 antibody variants with different functional CDRs (at LC and HC or germline) were generated by pRED Large Molecule Research at Roche Innovation Center Zurich and Munich.

### Study samples

Clinical serum samples were collected from the first-in-human, phase I cibisatamab monotherapy study (NCT02324257), an open-label, multicenter, dose escalation study. This study was approved by each center’s ethics committee or institutional review board and was conducted in conformance with the Declaration of Helsinki, the International Conference on Harmonization Guidelines for Good Clinical Practice, and appropriate laws and regulations. All enrolled participants supplemented written, informed consent.

### Biolayer interferometry analysis to characterize anti-ID binding

The binding properties of the anti-ID reagents (including ADA-positive controls) to cibisatamab or anti-CD3 domain variants were evaluated using biolayer interferometry (Octet). All steps of the analytics were defined (baseline, loading, association, and dissociation). The BLI baseline signal is established by calibrating the SA-sensor tip in an assay buffer (PBS, 0.5% RPLA1, 0.002% Bronidox) for 300 s (5 min). Streptavidin sensor tips were saturated with biotin-labeled cibisatamab or anti-CD3 variants. To establish baseline signal 2, the loaded sensor tip was incubated another 300 s (5 min) in the assay buffer. The kinetic rate constants were monitored by adjusting the association time when a saturated concentration of the binding partner approached the equilibrium-binding signal (~10–30 min). Dissociation time was at least 600 s (10 min).

### Pharmacokinetic assay to analyze active cibisatamab

To determine the concentration of cibisatamab in human serum, a serial sandwich enzyme-linked immunosorbent assay (ELISA) was developed and validated based on regulatory guidelines (20, 21). Purified and biochemically characterized anti-ID antibodies were specifically selected as capture and detection reagent tools to develop a target-binding-competent PK assay to measure active exposure. Here, critical conditions to avoid dissociation of the drug-ADA complexes (e.g., dilution or incubation times) were considered (4–6). In detail, capture anti-ID antibody (mAb<Id-mAb<H-CEA>>IgG-Bi), calibrators (cibisatamab) and diluted serum samples, detection anti-ID antibody (mAb<Id-mAb<H-CD3>>IgG-Dig), and anti-digoxigenin-POD are added serially to a streptavidin-coated microtiter plate (SA-MTP). Each reagent was incubated for no longer than 1 h on a MTP shaker at 500 rpm, and after each step, the MTP was washed three times. Finally, the immobilized immune complexes were analyzed via ABTS and HRP substrates and photometrically determined. The quantification of cibisatamab is performed by back-calculating the absorbance values using the corresponding calibration curve with a non-linear 4-parameter Wiemer-Rodbard curve fitting function.

### Anti-drug antibody assay to analyze ADA responses to cibisatamab

A bridging enzyme-linked immunosorbent assay (ELISA) was developed and validated to detect cibisatamab antibodies in human serum based on regulatory guidelines (22–24) and as described by Shankar (3). As a positive control, a mixture containing equimolar concentrations of two monoclonal antibodies (mAb<Id-mAb<CD3>>IgG; mAb<Id-mAb<CEA>>IgG) with similar binding features was used.

### Characterization of ADA-specific domain binding: domain-detection assay

The classical ADA bridging screening assay is not designed to identify a single drug-binding domain of ADA or to identify CDR-specific epitopes of ADA binding.

To distinguish between ADAs that bind to different domains, domain detection ELISA assays were developed for the detection of anti-CD3 antibodies or anti-CEA antibodies (**Supplementary Figure 2**), similar to previous approaches (9). Monoclonal anti-ID antibodies mAb<Id<CD3>> IgG or mAb<Id-mAb<CEA>>IgG were used as ADA-positive controls for corresponding domain detection assays. Capture antibody biotinylated cibisatamab, or anti-CD3 fab domain, or anti-CEA fab´2 domain, calibrators (ADA positive controls), along with detection antibody (digoxigenin-labeled cibisatamab) and POD-conjugated anti-digoxigenin Fab fragments, were added to a streptavidin-coated microtiter plate (SA-MTP). Each reagent was incubated for 1 h on an MTP shaker at 500 rpm, and after each step, the MTP was washed three times and residual fluids were removed. Next, the immobilized immune complexes were visualized by the addition of ABTS solution, an HRP substrate, which converted to a colored reaction product. Finally, the color intensity is photometrically determined, and the signal is proportional to the analyte concentration in the serum sample.

Similarly, to determine the binding of anti-ID antibodies directed to specific CDRs of the anti-CD3 domain, different engineered constructs with modified anti-CD3 binding sites were generated and used as biotinylated capture reagents. These constructs include fully functional HC and LC CDRs, functional HC CDRs but germline on LC, functional LC CDRs but germline on HC, or negative control germline LC and HC (with abrogated CD3e specificity and CD3e binding).

### ADA isotype determination by ELISA

To detect IgG-specific ADAs directed to cibisatamab, a specific drug-ADA-IgG complex assay was used as previously described (11, 12).

IgM-specific ADAs detection was performed as previously described (13). Here, cibisatamab has been biotin-labeled and bound onto an SA-MTP to capture IgMs.

### Cut point determination of ADA assays

CP determination for ADA bridging ELISA: The ADA screening and confirmatory cut points (SCP and CCP) were evaluated according to Shankar et al. by triplicate analysis of 100 individual samples collected from healthy volunteers (50 men and 50 women). Following pooling of all data points and exclusion of technical outliers, the remaining 299 data points showing a non-normal distribution were used to establish a screening cut point applying a 95th percentile leading to an expected false positive rate of 5% and a confirmatory cut point applying a 99th percentile leading to an expected false positive rate of about 1% (3). For the screening assay, an additive normalization factor was used to establish analytical run-specific cut points during sample analysis. Samples showing signals below the plate-specific SCP were rated as negative. Samples with signals equal to or above the plate-specific CP were rated as putatively positive and were reanalyzed in the confirmatory assay in the absence or presence of an excess drug concentration. Samples showing a signal inhibition equal to or greater than the CCP were deemed confirmed positive and were tittered.

CP determination for ADA Domain ELISA: The ADA domain-specific cut points were established as described before for the ADA screening assay by triplicate analysis of 40 healthy individual samples using either the anti-CD3 fab domain or the anti-CEA fab´2 domain of cibisatamab instead of the whole molecule as capture molecules, in combination with analyte-specific positive controls (monoclonal anti-idiotypic antibodies). Additive normalization factors were used to calculate analytical run-specific cut points.

CP determination for ADA Isotype Analysis: For this exploratory analysis, individual-specific cut points of patient serum samples were determined by averaging the OD values of every pre-dose sample of each patient and multiplying it by factor 2. These cut points were used for the evaluation of the study samples to reduce the effect of individual variations of baseline signals. Study samples with signals equal to or above the individual-specific cut point were rated as ADA IgG/IgM positive. Study samples with signals below the individual-specific cut point were rated as ADA-negative.

### Cell-based CD3ϵ receptor-reporter assay

The Jurkat NFAT reporter cells (Promega, Madison, WI, USA) were cultured at 0.1–0.5 Mio cells/mL in RPMI 1640 (Thermo Fisher Scientific, Waltham, MA, USA) containing HEPES, GlutaMax and additionally 10% FBS, 1 × NEAA, and 1x SoPyr (Thermo Fisher Scientific, Waltham, MA, USA). For the analysis, negative/positive control or samples were added in triplicate with a final serum concentration of 10% in white flat bottom 96-well plates. The plate was incubated for 4 h 45 min at 37°C, 5% CO2, brought to room temperature, and finally 35 µl of OneGlo Ex substrate were added. After 2 min of shaking at 1.000 rpm, the luminescence signals were measured on a Tecan Infinite F500.

## Data availability statement

The orginial contribution presented in the study are included in the article/**Supplementary Material**. Specific data availability statement to the clinical trial NCT02324257 has been published (25). Further inquiries can be directed to the corresponding author.

## Ethics statement

The studies involving humans were approved by each center’s ethics committee or institutional review board and was conducted in conformance with the Declaration of Helsinki, International Conference on Harmonization Guidelines for Good Clinical Practice, and appropriate laws and regulations. The studies were conducted in accordance with the local legislation and institutional requirements. The participants provided their written informed consent to participate in this study. Written informed consent was obtained from the individual(s) for the publication of any potentially identifiable images or data included in this article.

## Author contributions

GL: Conceptualization, Data curation, Formal Analysis, Investigation, Methodology, Supervision, Visualization, Writing – original draft, Writing – review & editing. AL: Data curation, Formal Analysis, Investigation, Methodology, Validation, Writing – review & editing. MM-F: Data curation, Formal Analysis, Writing – review & editing. AH: Data curation, Formal Analysis, Investigation, Methodology, Writing – review & editing. ES: Data curation, Formal Analysis, Methodology, Validation, Writing – review & editing. EM: Data curation, Formal Analysis, Methodology, Writing – review & editing. MA: Data curation, Formal Analysis, Writing – review & editing. TS: Data curation, Formal Analysis, Methodology, Writing – review & editing. MV: Data curation, Formal Analysis, Methodology, Writing – review & editing. RH: Data curation, Writing – review & editing. AF-G: Data curation, Formal Analysis, Methodology, Writing – review & editing. CK: Investigation, Supervision, Writing – review & editing. MS: Data curation, Formal Analysis, Writing – review & editing. MR: Methodology, Writing – review & editing. K-GS: Investigation, Resources, Supervision, Writing – review & editing.
