# Supplementary material for: Characterization of anti-drug antibody responses to the T-cell engaging bispecific antibody cibisatamab to understand the impact on exposure
Source: Front Immunol. 2024 May 31;15:1406353. doi: 10.3389/fimmu.2024.1406353 (PMC11176492; doi:10.3389/fimmu.2024.1406353)

Supplementary Material

**Supplementary Figure 1.** Development of a MoA based PK assay that is sensitive for ADA impact on exposure. (A) Schemata to illustrate ADA domain specific impact on cibisatamab exposure. (B) Monocloncal anti-ID ADA positive controls (PC) show similar binding profiles to cibisatamab using biolayer interferometry (sensograms). (C) Using an equimolar mix of ADA PC anti-Id <<CEA>> and anti-Id <<CD3>>, the recovery of cibisatamab (120 ng/ml) were measured using MoA-based PK assay without or with increasing concentration of the ADA PC mix (graph).


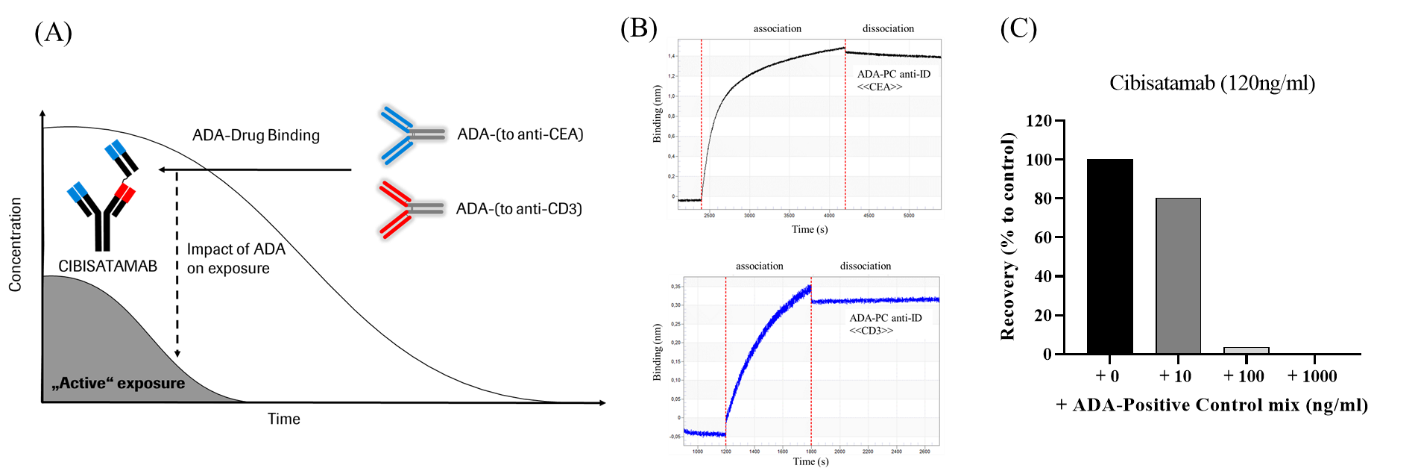


**Supplementary Figure 2:** Schemata to illustrate assay format of the domain specific detection. Cibisatamab based ADA bridging ELISA (top) were used as screening ADA assay.


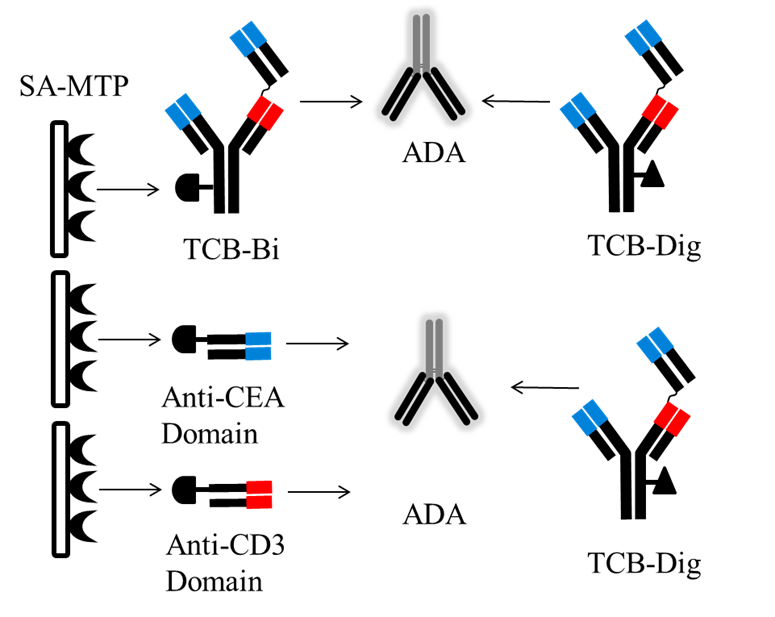
Supplementary Figure 3: Characterisation of ADA isotypes and its kinetics along with exposure and titer.


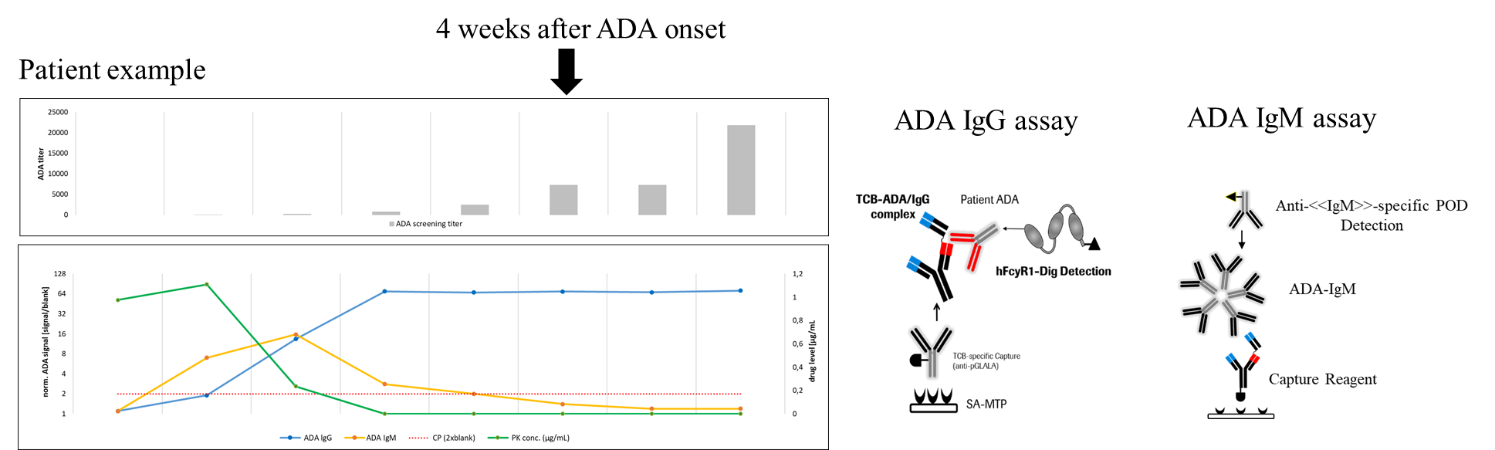


**Supplementary Figure 4**: Binding characteristics of four different monoclonal anti-idiotypic ADA positive controls to anti-CD3 domain. (A) Anti-ID binding to cibisatmab analyzed via ELISA (B) Anti-ID binding to cibisatamab analyzed via biolayer-interferometry.


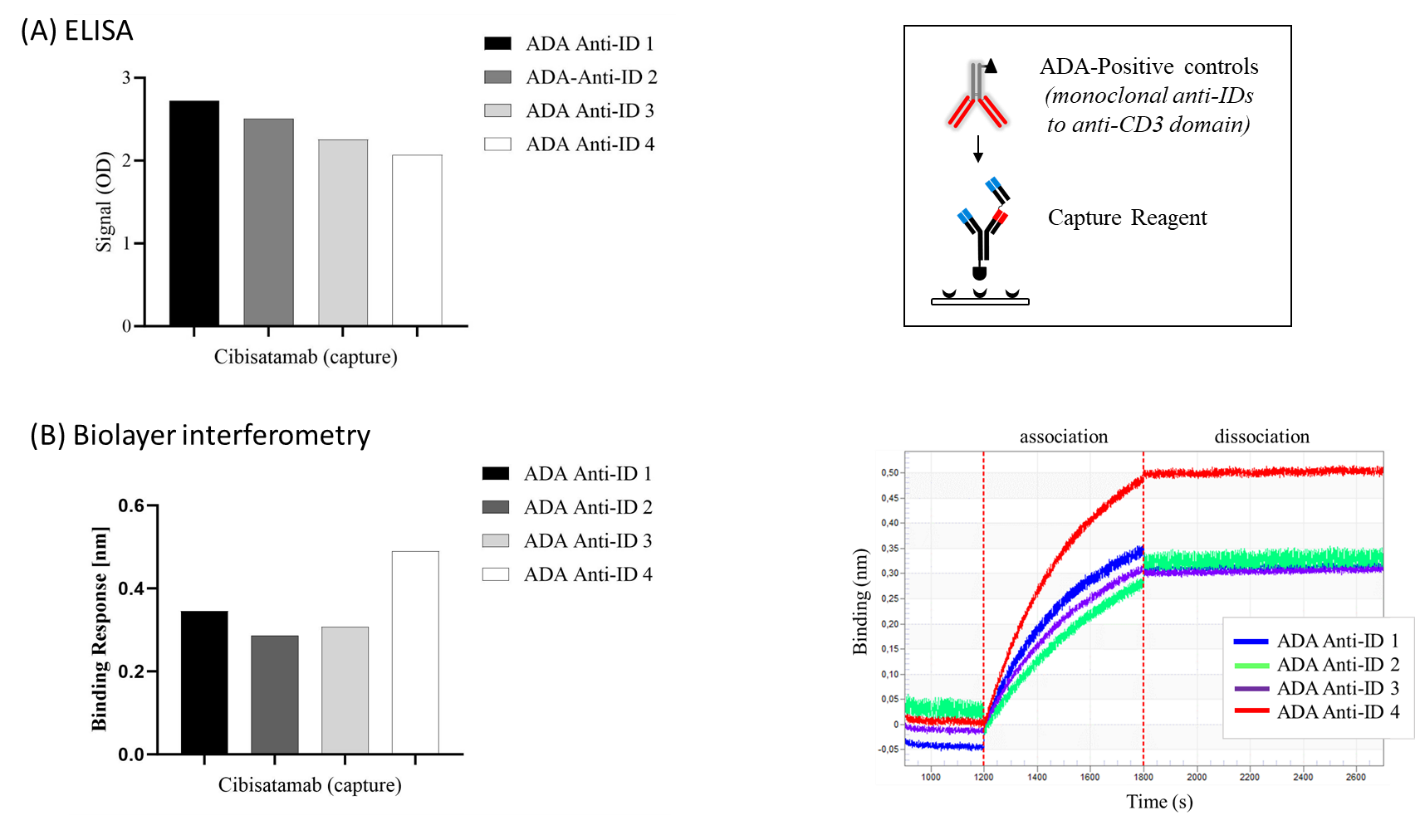


**Supplementary Figure 5**: Specific anti-CD3 domain constructs were engineered, purified, and implemented as capture reagents for the detection of ADAs directed to CDRs of the anti-CD3 domain of cibisatamab. Labelled cibisatamab was used as detection reagents.


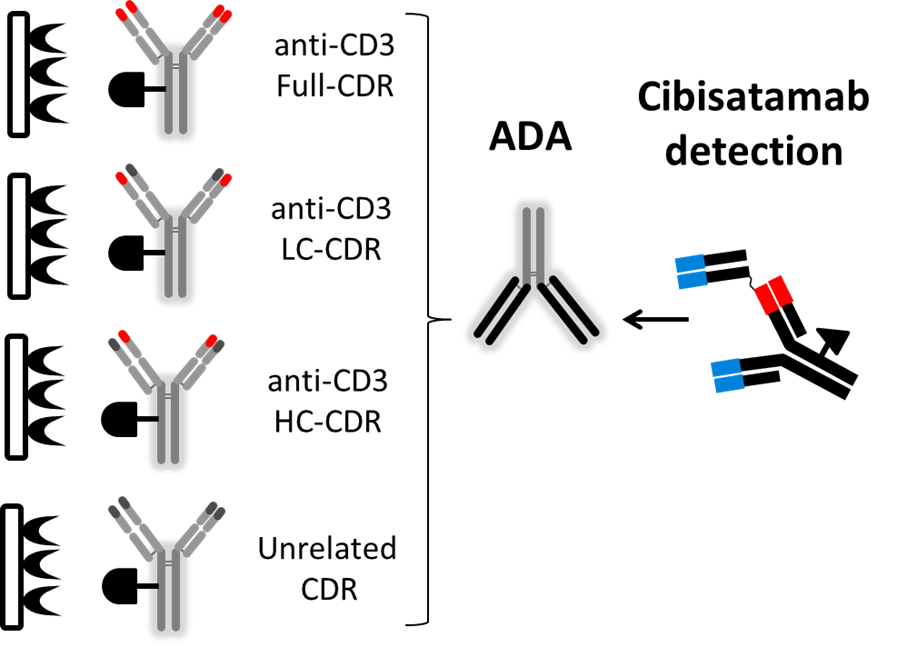

Supplement: Supplementary file 1 [file DataSheet_1.docx]
